# Learning from recent outbreaks to strengthen risk communication capacity for the next influenza pandemic in the Western Pacific Region

**DOI:** 10.5365/wpsar.2018.9.5.013

**Published:** 2019-02-19

**Authors:** Lauren J O’Connor, Lisa Peters, Rose Aynsley

**Affiliations:** a WHO Regional Office for the Western Pacific, Division of Health Security and Emergencies.; b WHO Regional Office for the Western Pacific, Division of Pacific Technical Support.

When an influenza pandemic swept the globe in 1918, it was nicknamed the “Spanish flu” despite evidence of circulation in other countries. This was because the Spanish press were free to publish stories about the outbreak that peers in neighbouring countries were not due to wartime censors. (*1*) Other governments hid negative news about the pandemic and over-reassured the public. Attempts to prevent panic backfired, and the resulting breakdown in trust “threatened to break the society apart.” (*1*)

The 1918 pandemic illustrates the consequences of failing to transparently and effectively communicate risks to the public during a public health event. This article discusses the lessons learnt in risk communication during the response to recent outbreaks in the World Health Organization’s Western Pacific Region. These lessons can inform preparedness for pandemic influenza and other public health threats.

Risk communication is defined as “the real-time exchange of information, advice and opinions between experts, community leaders, or officials and the people who are at risk.” (*2*) The outbreak of severe acute respiratory syndrome (SARS) in China in 2002 in particular highlighted the importance of open risk communication – a lesson that was reiterated once more during the outbreak of Middle East respiratory syndrome in the Republic of Korea in 2015. Effective risk communication during a public health emergency can be difficult, especially in the early stages when many of the facts may be uncertain. Health authorities can be reluctant to proactively communicate as they are apprehensive of saying the wrong thing, creating panic or looking like they do not have all the answers. However, delaying communication can result in the public listening to rumours or relying on less accurate sources of information, (*3*) or can lead to the very panic authorities were trying to prevent. (*4*)

Done correctly, however, risk communication can calm fears, facilitate the acceptance of containment measures, curtail the spread of unhelpful rumours and engage affected communities in control measures. In the wake of SARS, risk communication was included as a core capacity required of Member States under the International Health Regulations (2005). (*5*) Guidance on how to implement and build risk communication capacity has also been part of the Asia Pacific Strategy for Emerging Diseases (APSED) since the first 2005 edition. (*6*) It has long been recognized that national risk communication plans, supported by trained risk communication personnel, adequate financial allocations, clear internal procedures and mechanisms for coordination, are essential for effective risk communication and should be established before the onset of a public health emergency. Advance preparation, including building an understanding of prevailing cultural practices and establishing relationships with community influencers, is central to ensuring that risk communication efforts are tailored to the local context.

The 2009 influenza A(H1N1) pandemic highlighted some further lessons that apply specifically to influenza and need to be considered ahead of the next influenza pandemic. For example, perceptions of the severity of the disease varied widely. Many people confused it with seasonal influenza and therefore thought the risk to their health was low, while others expressed a high level of concern about the virus. (*7*) Some people also had immense distrust in the vaccine, because of perceived conflicts of interest between pharmaceutical companies and health authorities. (*7*) Research published following the H1N1 pandemic indicated that countries should be prepared to address rumours and misconceptions about vaccine safety, to carefully communicate the severity of disease and to enlist the support of trusted members of the community. (*7*) The role of social media also needed to be considered. (*8*) After the 2009 pandemic, it was recognized that risk communication approaches could be tested and honed during seasonal influenza outbreaks. (*9*)

Ten years after SARS, China proactively informed the public and international community about human cases of avian influenza (H7N9), demonstrating the benefit of timely and transparent risk communication. (*10*) Chinese focus group participants were reassured by this increase in transparency, with one participant stating, “I am quite positive that our government absolutely has the capability to control this disease.” (*11*) Another study found that discussion of H7N9 on Sina Weibo, a popular Chinese social media platform, dropped following several formal announcements, potentially indicating reduced public concern about the outbreak. (*10*)

While social media was used to listen to the public following the discovery of human cases of H7N9, the response to an outbreak of influenza-associated severe acute respiratory infections (SARI) in Fiji in 2016 showed that more traditional means of communication still have a place in effective risk communication. Health authorities worked with religious leaders, women’s groups and youth networks to engage vulnerable groups, particularly pregnant women, encouraging vaccination and adoption of protective behaviours. (*12*, *13*) For communities in remote areas and outer islands, where communication is often limited, authorities shared health messages via radio, reaching an estimated 90% of the population. (*14*)

Unfortunately, while much progress has been achieved in risk communication under APSED, other core public health capacities for pandemic preparedness and response continue to be prioritized over risk communication. Results from joint external evaluations of IHR core capacities in the Western Pacific Region show that countries score far higher on traditional public health capacities, such as surveillance and laboratory networks, than they do on risk communication. (*15*) Countries are encouraged to learn from recent outbreaks and emergencies and to invest in their internal capacity for risk communication as per the Asia Pacific Strategy for Emerging Diseases and Public Health Emergencies (APSED III). (*16*) This includes integrating risk communication into outbreak preparedness, planning and response, communicating quickly and transparently, using a mixture of channels to best reach their target audience (including social media, where appropriate) and actively engaging communities in the response.

The vision laid out in APSED III is one where risk communication moves from being purely an art to also a science, as risk communication becomes more professionalized and evidence-based. Risk communication professionals should come to be recognized as social scientists conducting work that is as important to the success of emergency preparedness and response as the work of epidemiologists, laboratory experts and other public health personnel. In prioritizing and strengthening risk communication, countries will be better placed to limit the health, social and economic impacts of the next influenza pandemic.

## References

[1] Barry J. The great influenza, the epic story of the deadliest plague in history. New York (NY): Viking Books; 2004.

[2] Communicating risk in public health emergencies: A WHO guideline for emergency risk communication (ERC) policy and practice. Geneva: World Health Organization; 2017 (https://apps.who.int/iris/bitstream/handle/10665/259807/9789241550208-eng.pdf?sequence=2, accessed 23 March 2018). 31059213

[3] Fung ICH, Tse ZTH, Chan BSB, Fu KW. Middle East respiratory syndrome in the Republic of Korea: transparency and communication are key. West Pac Surveill Response. 2015 Aug 7;6(3):1–2. doi:10.5365/wpsar.2015.6.2.011 pmid:26668758. 10.5365/wpsar.2015.6.2.011 PMC4675162 26668758

[4] Huang Y. The SARS epidemic and its aftermath in China: a political perspective. In: Institute of Medicine; Knobler S, Mahmoud A, Lemon S, et al., editors. Learning from SARS: preparing for the next disease outbreak: workshop summary. Washington, DC: National Academies Press; 2004 (https://www.ncbi.nlm.nih.gov/books/NBK92479/, accessed 2 April 2018). 22553895

[5] International Health Regulations. (2005). Third edition. Geneva: World Health Organization; 2016 (http://apps.who.int/iris/bitstream/handle/10665/246107/9789241580496-eng.pdf;jsessionid=911704AF9CB220DFF2729886EDF9FEEA? sequence=1)

[6] Asia pacific strategy for emerging diseases (2005). Manila: WHO Regional Office for the Western Pacific; 2005 (https://iris.wpro.who.int/bitstream/handle/10665.1/14080/9290612096-eng.pdf)

[7] Barrelet C, Bourrier M, Burton-Jeangros C, Schindler M. Unresolved issues in risk communication research: the case of the H1N1 pandemic (2009-2011). Influenza Other Respir Viruses. 2013 9;7 Suppl 2:114–9. 10.1111/irv.12090 24034495 PMC5909386

[8] Itzwerth R, Moa A, MacIntyre CR. Australia’s influenza pandemic preparedness plans: an analysis. J Public Health Policy. 2018 2;39(1):111–24. 10.1057/s41271-017-0109-5 29176589

[9] 2009 H1N1 influenza improvement plan. Washington, DC: US Department of Health and Human Services; 2012 (https://www.phe.gov/Preparedness/mcm/h1n1-retrospective/Documents/2009-h1n1-improvementplan.pdf, accessed 26 March 2018).

[10] Vong S, O’Leary M, Feng Z. Early response to the emergence of influenza A(H7N9) virus in humans in China: the central role of prompt information sharing and public communication. Bull World Health Organ. 2014 4 1;92(4):303–8. 10.2471/BLT.13.125989 24700999 PMC3967572

[11] Li R, Xie R, Yang C, Frost M. Perceptions on the risk communication strategy during the 2013 avian influenza A/H7N9 outbreak in humans in China: a focus group study. West Pac Surveill Response. 2016 7 11;7(3):21–8. 10.5365/wpsar.2016.7.1.005 27757257 PMC5053133

[12] Collins J, Biaukula V, Faktaufon D, Flint J, Fullman S, Jalava K, et al. An outbreak investigation of paediatric severe acute respiratory infections requiring admission to intensive care units - Fiji, May 2016. West Pac Surveill Response. 2018 6 21;9(2):4–8. 10.5365/wpsar.2017.8.4.009 30057852 PMC6053106

[13] Analysis FAHS. Suva: Fiji Ministry of Health and Medical Services; 2016 (https://www.aidsdatahub.org/sites/default/files/Fiji_Adolescent_Health_Situational_Analysis_2016.pdf, accessed 29 October 2018).

[14] Health and Nutrition Cluster bulletin #8. Suva: Fiji Ministry of Health; 2016 (http://www.health.gov.fj/wp-content/uploads/2016/03/20160613_HNC_Bulletin8_final.pdf)

[15] Western Pacific Region WHO. JEE mission reports. Geneva: World Health Organization; 2018. [cited 2018 November 2]. Available from: https://www.who.int/ihr/procedures/mission-reports-western-pacific/

[16] Asia Pacific strategy for emerging diseases and public health emergencies (APSED III). Manila: WHO Regional Office for the Western Pacific; 2017 (https://iris.wpro.who.int/bitstream/handle/10665.1/13654/9789290618171-eng.pdf, accessed 5 November 2018).

